# Factors Affecting Hospital Provision of Health-Promoting Services Transferred to Provincial Administration in Thailand

**DOI:** 10.3390/ijerph21081053

**Published:** 2024-08-12

**Authors:** Atcharawadee Sriyasak, Chaweewan Sridawruang, Pennapa Sriring, Bandit Nitkhamhan, Manaporn Chatchumni, Boonruang Khaonuan, Atiya Sarakshetrin

**Affiliations:** 1 Faculty of Nursing, Praboromarajchanok Institute of Health Workforce Development, Boromarajonani College of Nursing, Yala 95000, Thailand; atcharawadee@bcnyala.ac.th; 2 Faculty of Nursing, Praboromarajchanok Institute of Health Workforce Development, Boromarajonani College of Nursing, Khonkaen 40000, Thailand; 3 Faculty of Public Health and Allied Health and Sports Science, Sirindhorn College of Public Health Khonkaen, Thaksin University, Khonkaen 40002, Thailand; pennapa@scphkk.ac.th (P.S.); bandit@scphkk.ac.th (B.N.); 4 School of Nursing, Rangsit University, Pathumthani 12000, Thailand; 5 Faculty of Health and Sports Science, Thaksin University, Phatthalung 93210, Thailand; bkhaonuan@gmail.com; 6 Faculty of Nursing, Praboromarajchanok Institute of Health Workforce Development, Nonthaburi 11000, Thailand; atiya_s@hotmail.com

**Keywords:** service delivery policy, primary healthcare units, healthcare service quality, nursing competencies, health system transition

## Abstract

Following the transfer of hospital services to provincial government oversight, it is essential to understand how such changes impact service quality and efficiency. This study, conducted from 1 March 2023 to 31 March 2024 at sub-district health-promoting hospitals in Thailand, aims to identify the factors affecting hospital service quality post-transfer and evaluate the role of professional nurses in maintaining service standards under new governance. Utilizing a mixed-methods design based on the Global Fund-HSS framework (2012), which categorizes factors into accessibility, processes, productivity, and outcomes, we collected data from 340 nurses and 400 patients through structured questionnaires. These questionnaires were divided into seven sections, capturing metrics such as the personal data of nurses, hospital service activities, and leadership qualities. We employed a Likert scale to gauge the perceptions and expectations of service quality and conducted paired *t*-tests to compare performance metrics before and after the administrative transfer. One-way ANOVA was used to assess variability among different service units, and binary logistic regression helped identify the key determinants of service quality. The analysis revealed a significant correlation between the attitudes and competencies of healthcare teams and the levels of service quality. Notably, service units transferred less than 50% exhibited minimal changes, indicating that the degree of transfer significantly impacts service outcomes. Despite these variations, the fundamental mission of promoting health remained consistent. The study emphasizes the critical role of nurses and recommends further research to identify additional factors that could improve service quality in transferred healthcare facilities, potentially contributing to enhanced healthcare delivery across restructured health systems.

## 1. Introduction

Thailand recognizes healthcare decentralization as a key strategy for enhancing the quality, efficiency, and equity of its health systems. This approach was facilitated by the 1997 Constitution [1,2,3], which empowered local authorities and citizens to manage their health services. Subsequent legislation in 1999 and decentralization plans in 2000 and 2008 further outlined the processes for local governance to improve public health and disease management [1,2,3]. This restructuring extends to various local administrative bodies, underscoring the role of decentralization in supporting effective health insurance and overall healthcare provision at the local level [4].

According to the Thai Constitution of 2017 [4], the decentralization of public health responsibilities aims to empower local governance to manage and provide health services in accordance with legal stipulations. This includes the planned transfer of responsibilities to local governing bodies for public health services and activities, as well as defining processes for transferring responsibilities, budgets, and personnel [4]. National reform policies provide guidelines and procedures for transferring and appointing committees to manage new responsibilities. Despite these measures, evaluations from 2008 to 2020 revealed delays in the transfer process, with only 60 to 70 health-promoting hospitals being transferred out of a total of 9787 across the country. [4]. The appointment of committees to oversee these transfers has also not met planned targets, necessitating a review and improvement of the transfer processes to ensure efficient and effective public health operations according to reform policies [1,2,3,4].

Primary care services are critical for national public health development, emphasizing continuous quality access to healthcare. Robust primary healthcare systems, as shown by international data analysis, enable efficient access to essential health services. Local health committees have designated health-promoting hospitals for management and integration within the health system, aiming to enhance service quality and benefit the local population [5,6].

Developing primary healthcare systems is crucial for ensuring equitable access to health services and reducing hospital congestion in Thailand [7,8,9,10,11]. In 2023, new health service standards were issued to foster development and evaluate service quality equitably and efficiently [11]. However, issues related to social and cultural standards persist, where healthcare personnel behavior plays a significant role. It is important, therefore, to focus on developing primary healthcare personnel through training and skill development in clinical nursing to enhance patient care, health promotion, and disease prevention [12,13,14,15,16,17]. Moreover, primary care units need improvement in service integration and human resource management [18,19]. Problems such as lack of staff motivation, inadequate medical supplies, unlinked referral systems, and insufficient funding have emerged following the transfer of health-promoting hospitals to local government organizations, resulting in decreased service quality [20].

Given these challenges, this study aims to investigate the factors affecting hospital service quality post-transfer and evaluate the critical role of professional nurses in maintaining service standards under new governance. Specifically, the research questions guiding this study are as follows:

1. What are the main factors influencing the quality of service in health-promoting hospitals following their transfer to local governance?
2. How do healthcare teams’ attitudes and competencies correlate with service quality levels in these decentralized settings?

By focusing on sub-district health-promoting hospitals and employing a mixed-methods design, this study seeks to provide insights into how decentralization has impacted the operational efficiency and service quality of healthcare facilities. The significance of this research lies in its potential to inform policy adjustments and practical improvements, thereby enhancing healthcare delivery within Thailand’s restructured health system. The findings are intended to support further refinements in policy and practice, addressing the gap between decentralization policy intentions and their practical implementation.

## 2. Materials and Methods

### 2.1. Conceptual Framework

The study employs the Global Fund-HSS framework [19], which is organized into four major sections to comprehensively assess the factors influencing healthcare service quality and efficiency.

#### 2.1.1. Input Factors

This section includes six building blocks focusing on service delivery and the workforce. It encapsulates all resources used to deliver health services, such as infrastructure, medical equipment, and human resources, highlighting the foundational elements that contribute to healthcare operations.

#### 2.1.2. Processes

Derived from nursing skills, this segment encompasses treatment, promotion, prevention, and rehabilitation processes. It examines how healthcare services are implemented on the ground, focusing on the methodologies and practices used by nursing professionals to deliver care.

#### 2.1.3. Outputs

This component covers the services provided in healthcare facilities and communities. It measures the direct results of healthcare processes, assessing how effectively health services are delivered and received in both hospital and community settings.

#### 2.1.4. Outcomes

Guided by SERVQUAL and the QOF [4,13], this section measures the effectiveness and quality of the healthcare services provided. SERVQUAL helps in assessing service quality through multiple dimensions such as reliability, assurance, tangibles, empathy, and responsiveness, while the QOF evaluates the outcomes based on predetermined quality indicators.

**Figure 1:**
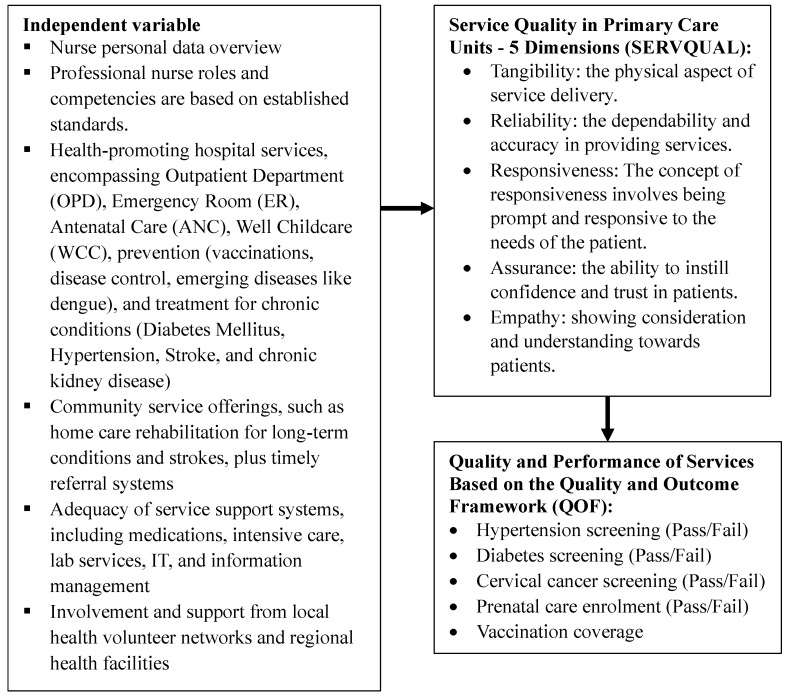
Research concept framework.

Integrating the Global Fund-HSS framework with the SERVQUAL and QOF models enables a comprehensive analysis of both the functional aspects of healthcare delivery and the perceived quality of services from patient and provider perspectives. This methodological approach not only captures a wide range of healthcare dynamics but also facilitates a detailed evaluation of the impacts of decentralization on service quality and efficiency. (Figure 1).

This mixed-methods study was conducted from 1 March 2023 to 31 March 2024 at Hua Hin Hospital in Thailand, which was selected for its active involvement in health-promoting services under the new provincial administration model in Thailand. The research project was presented and approved by the Ethics Committee for Research in Human Subjects at Hua Hin Hospital, project number RECHHHNo.011/2023. The hospital’s selection was based on its exemplary implementation of health-promoting initiatives, aligning with the study’s focus on evaluating ‘Factors Affecting Hospital Provision of Health-Promoting Services Transferred to Provincial Administration in Thailand’. The study was carried out according to the established procedures. Researchers explained the objectives, expected benefits, methods, and procedures that participants were required to follow, ensuring the confidentiality of the collected data, which was anonymized to be used solely for research purposes. Participants had the right to refuse or withdraw from the study without any consequences. Consent was obtained from all participants who signed the informed consent form before participating in the study.

This section is divided by subheadings. It provides a concise and precise description of the experimental results, their interpretation, as well as the experimental conclusions that can be drawn.

### 2.2. Population and Sample

The sample consisted of 342 professional nurses, calculated using Daniel and Cross’s formula [12], resulting in a sample size of approximately 341 persons.

$$n=\frac{3263\;x3.841x0.55x0.45}{\left(0.05{)}^{2}(3266-1)+3.841(0.55x0.45\right)},\;n=340.7.$$

Eligible participants in this study were professional nurses with at least six months of experience in health-promoting hospitals following their transition. To prioritize participant well-being, we established discontinuation criteria, including provisions for terminating data collection and securely destroying any personal documents if participants experience discomfort or distress from the research questions. Furthermore, any instances of coercion by superiors to participate resulted in the immediate cessation of data collection and the secure destruction of related documents. Structured questionnaires collected data from 340 nurses and 400 patients. In cases of participant discomfort or distress from the study questions, data collection will be terminated immediately, and all personal documents will be securely destroyed. Should any reports of coercion by superiors emerge, the same immediate actions will be taken to ensure participant safety and data integrity. This data analysis allowed for the classification of factors affecting the provision of health-promoting services into four key domains: accessibility, processes, productivity, and outcomes, aligning with the objectives of this study.

### 2.3. Research Instruments

The study used a questionnaire with seven sections with eight items. Part 1 includes general personal information about nurses. Part 2 covers the health-promoting hospital’s general information and activities. Part 3 concerns the service provider team. Part 4 focuses on core indicators (Quality and Outcome Framework: QOF) and primary service outcomes, which includes the percentage of Thai population aged 35–74 screened for diabetes by blood sugar level. We screened a portion of the Thai population aged 35–74 for high blood pressure. The study aims to determine the percentage of pregnant women who receive their first antenatal care within 12 weeks, as well as the percentage of women aged 30–60 screened for cervical cancer within a five-year period. Part 5 is a questionnaire with 39 questions about the roles, duties, and skills of professional nurses in hospitals that promote health. The questions are based on the Canadian Nurses Association’s studies [13] on the roles, duties, skills, and scope of practice of professional nurses, as well as other relevant studies [13]. Part 6 rates the quality of service in primary care units based on how people actually feel and what they expect. It does this by using a questionnaire created by Kittisak Saengthong [15] that uses the five dimensions of SERVQUAL: tangibility, reliability, responsiveness, assurance, and empathy. Each dimension has five questions, and the answers are given on a five-point Likert scale [17]. Part 7 contains 11 items about leadership qualities necessary for administrators in health-promoting hospitals and is scored on a five-point Likert scale, calculating average scores per Best’s formula [14].

**Figure 2:**
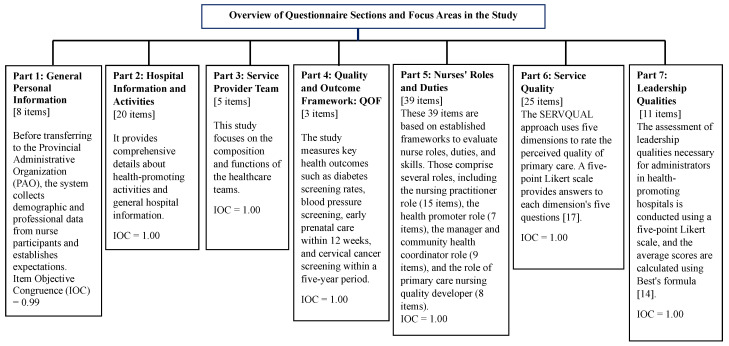
Structure of the questionnaire [15].

The study includes Figure 2 below to better illustrate the comprehensive nature and structure of the questionnaire. This figure visually depicts the seven sections of the questionnaire, highlighting the main focus of each part and the specific health metrics evaluated. The design of each section gathers distinct yet interconnected types of data, which collectively contribute to a holistic evaluation of the health-promoting services provided by the hospitals under study.

### 2.4. Quality Verification of Research Instruments

Three qualified experts assessed the content validity of the research instruments, leading to revisions based on their recommendations. This included calculating the Index of Item Objective Congruence (IOC) [12] for seven parts of the nursing research questionnaire, yielding indices of 0.99, 1.00, 1.00, 1.00, 1.00, 1.00, and 1.00, respectively.

### 2.5. Data Collection

Preparation Stage: The researcher, a crucial tool in data collection and analysis, has extensive experience conducting and publishing both domestic and international qualitative research. Initial steps involved establishing contact and inviting participants, clearly explaining the research objectives and scope. Implementation Stage: The researcher-built rapport with participants by introducing themselves and explaining the purpose and details of the questionnaire. The researcher scheduled appointments for data collection, specifying the place, date, and time. Data Collection: The researcher collected data in private settings. The researcher minimized bias and maintained consistent conduct to underline the importance of the data collected, ensuring no significant data were overlooked.

### 2.6. Data Analysis

Descriptive Statistics: To summarize the data, we conducted descriptive statistical analyses of the sample group’s personal data using SPSS version 26.0 (IBM Corp., Armonk, NY, USA). To assess the effectiveness of the intervention before and after its implementation, we applied the paired *t*-test. For comparing effectiveness across different groups, we employed the one-way ANOVA technique. Additionally, we utilized binary logistic regression analysis to investigate the factors influencing the quality of service provision. We performed all these analyses using the aforementioned version of SPSS, ensuring consistency and reliability in our statistical approach.

We conducted the qualitative data analysis using Streubert-Speziale and Carpenter’s [21] content analysis guidelines. The process included the following. (1) Understanding the data: the researcher read and reviewed each line of the transcribed interview data to grasp an overall understanding of the participants’ thoughts, feelings, and experiences, keeping the research objectives in mind. (2) Meaning units: we identified words, sentences, or paragraphs as important meanings reflecting the participants’ experiences related to primary care service quality [21]. We then condensed these into condensed meaning units, coded them, and organized them into related sub-themes and broader themes. (3) Linking themes: the final step was to connect the sub-themes to form comprehensive themes that reflect the quality of primary care services.

### 2.7. Ensuring Trustworthiness

Lincoln and Guba [21] established the trustworthiness of the data through several methods. Expert reviews and participant confirmation ensured credibility by confirming the coverage, depth, and saturation of the data. The researcher achieved reliability by independently analyzing reflection notes and group discussion transcripts, then collaborating with co-researchers and community and qualitative research experts to review the findings. The researcher addressed transferability by selecting a diverse sample group that varied by age, gender, and study area, thereby providing data applicable to similar contexts. We maintained confirmability throughout the study by triangulating data using various documents, including audio recordings, field notes, and personal reflection journals, analyzed in NVivo 10 [22], to ensure consistent reference and verification of the data.

## 3. Results

### 3.1. Overview of Services at Sub-District Health-Promoting Hospitals under Provincial Administrative Organizations

As mandated by the Primary Health System Act of 2019, the majority of medium-sized hospitals in Health Area 7 were categorized as PCUs (Primary Care Units), with 59.82% recognized as PCUs and 32.54% as NPCUs (Non-Primary Care Units) before being transferred to the provincial administrative organization. Initially, these hospitals enjoyed a high star rating of 98.83%; however, this rating declined to 83.63% subsequent to the transfer. On average, these hospitals serve 5104.05 individuals (SD = 3504.05), predominantly serving areas with 3000 to 8000 people (60.69%) and managing no more than 1000 households (63.11%). The coverage extends to health insurance (54.60%), civil servant benefits (69.84%), and social security (54.81%), with responsibilities spanning 6–10 villages (47.48%). Research indicates variability in the roles and competencies of professional nurses’ post-transfer. The highest recorded competency was in health promotion, with an average score of 4.15 (SD = 0.66). In contrast, the competency in developing primary nursing quality was the lowest, averaging 3.76 (SD = 0.81). Specific tasks such as patient history-taking and assessment showed consistency, each scoring 4.41 (SD = 0.62 and SD = 0.66, respectively).

### 3.2. Factors Affecting Service Provision at Sub-District Health-Promoting Hospitals Transferred to Provincial Administrative Organizations

The analysis focused on service quality in primary care units. (1) The analysis measured service provision across six aspects: medical treatment, health promotion, disease prevention and control, rehabilitation, emergency care, and palliative care management. Post-transfer, the highest average scores were notably within the group that transferred entirely, especially in tangible aspects of service at a mean of 3.92, reliability at 4.28, responsiveness at 4.19, assurance at 4.40, and empathy at 4.61. (2) Expectation vs. reality post-transfer: when comparing expectations to actual perceptions of service post-transfer, the expectation scores were consistently higher than the actual perception scores across all aspects, showing statistically significant differences at the 0.05 level. This discrepancy was particularly pronounced in the group with 100% transfer, highlighting significant statistical differences across all aspects at the 0.05 level. In contrast, groups with less than 50% transfer exhibited higher expectation scores than perception scores, with significant differences only in the dimensions of equitable and discrimination-free service provision, and no significant statistical differences in attentive and respectful treatment. (3) Staff attitudes before and after transfer: before the transfer in the fiscal year 2022, the overall average attitude score of the service team was 3.60 (SD = 0.88), with the highest average concerning patient and public benefit at 4.21 (SD = 0.67). Post-transfer in 2024, the overall average improved slightly to 3.72 (SD = 0.93), maintaining the highest scores for patient and public benefit at 4.21 (SD = 0.77) and improved relations with colleagues at 4.04 (SD = 0.73). The aspect with the lowest average both before and after the transfer was compensation perceived as adequate, scoring 3.01 (SD = 0.84) initially and slightly increasing to 3.04 (SD = 1.01) after the transfer.

The outcome comparison across primary healthcare units’ indicators before and after a complete transfer indicated a significant reduction, particularly in the indicators affecting the overall population, as evidenced by statistical significance at the *p*-value of 0.05. In the cohort transferred between 50% and 99%, various indicators exhibited declines from the baseline, although some, like cervical cancer screenings for women aged 30–60 within five years, did not meet the statistical significance threshold. Notably, in units transferred less than 50%, significant variations were observed in health screenings such as diabetes and hypertension among the Thai population aged 35–74, underscoring the profound effects of service transfer on local health maintenance and development. These findings highlight the necessity of strategic interventions aimed at improving health outcomes, as outlined in Table 1, which presents nurses’ perspectives on services at sub-district health-promoting hospitals.

We employed ANOVA statistics in the analysis, stratified by the extent of transfer—100% in Group 1, 50–99% in Group 2, and less than 50% in Group 3—to assess the outcome differences across primary healthcare units. The analysis included the following key health indicators: diabetes screening among Thais aged 35–74, initial antenatal visits within 12 weeks for pregnant women, and cervical cancer screenings within 5 years for women aged 30–60. The findings revealed statistically significant variations in at least one of these groups, demonstrating differences in healthcare delivery effectiveness across the different levels of transfer that were significant at a *p*-value of 0.05, as shown in Table 2.

## 4. Discussion

The operational process of the study examining factors influencing service provision in sub-district health-promoting hospitals transferred to provincial administrative organizations can be divided into four systems: input, process, output, and outcomes, focusing on service outcomes and quality according to SERVQUAL and QOF. The discussion can be divided into three areas: (1) system development, (2) nursing practices, and (3) quality of care.

### 4.1. System Development

In the broader context of system development within primary care units, our study revealed that these units achieved the set goals across all indicators, with statistically significant differences at the 0.05 level in the transfer groups. Notably, the 100% transfer group demonstrated definitive outcomes, with average results for all indicators exceeding 90%. The provincial administrative organization (PAO) indicates that the changes in primary healthcare, aligned with public health policies and primary healthcare service standards [2,3,4], have effectively driven improvements with a focus on equity and efficiency. When examining specific tasks within this framework, our findings highlight a consistent performance in patient history-taking and assessment, each scoring 4.41 (SD = 0.62 and SD = 0.66, respectively). Acknowledging these consistencies is crucial, as they reflect the reliability of clinical practices in the face of systemic changes and can guide the ongoing refinement of community health services.

Furthermore, the study identified key factors influencing service provision, particularly decision-making and access to basic healthcare. Nursing played a significant role in the post-transfer development of the primary healthcare system. However, we noted the greatest discrepancies from expectations, including 81.87% progress in job functions, a decrease from expectations, and a 53.80% reduction in indicators, primarily due to staff shortages, insufficient medical supplies, unlinked referral systems, and inadequate budgeting. Also, people were less able to take care of themselves and behaved badly while receiving help [6]. This is similar to what Peerpun P. and Pasunon P. [23] found when they looked at problems with service delivery at a sub-district health-promoting hospital in Uttaradit Province. They reported issues such as inadequate service points, insufficient medical staff, and unstructured service processes, including a lack of consultation points and unclear community network participation. To overcome these barriers to access, strategies should encompass improvements to the service environment, augmentation of medical staff, integration of various health services, enhancement of consultation channels and information availability to the public, and the development of caregiver capabilities and community cooperation [23].

### 4.2. Nursing Practices

The study found that the majority of service recipients had high expectations and perceptions of quality response, with 97.50% and 89.75%, respectively. The factors related to accessibility and the capabilities of nurses in sub-district health-promoting hospitals post-transfer, with the highest average competency being the role in promoting health at an average of 4.15 (SD = 0.66) and the lowest in developing primary nursing quality at 3.76 (SD = 0.81). This study highlights the complexities and possibilities in the interaction between cultural beliefs, information needs, and practical and logistical obstacles faced by health providers, such as supply procurement and client needs [18]. Previous studies laid the foundation for developing intervention strategies to overcome access barriers by identifying and analyzing supporting factors that enable health providers to meet individual patient needs. Nurses’ expertise is critical; therefore, enhancing the development of health personnel within the primary care system is key to setting standards and augmenting nurse capabilities. This ensures the provision of high-quality, efficient healthcare services. Specifically, addressing the complex needs of patients with comorbidities requires targeted educational interventions. Referencing Lawson et al. [24], we propose incorporating an international comorbidity education framework that outlines structured educational strategies to enhance nurses’ competencies in this area. This framework suggests a multilayered approach to education, including updated clinical guidelines, scenario-based learning, and interprofessional collaboration, tailored to the needs of nurses handling comorbid conditions. This approach not only aligns with the general experiences and daily needs of service recipients [19,25] but also directly addresses the gaps in current training for managing comorbidities, thereby potentially improving patient outcomes and care efficiency.

### 4.3. Quality of Care

The study specifically analyzed outcomes in primary care units before and after the transfer of services, assessing quality service provision based on SERVQUAL and QOF criteria. We recorded a notable decline in outcomes post-transfer for the group that experienced a 100% transfer, and these differences were statistically significant at a *p*-value of 0.05. Furthermore, our analysis, as detailed in Table 2, specifically focused on critical health indicators: diabetes screening among Thai individuals aged 35–74, initial antenatal visits within 12 weeks for pregnant women, and cervical cancer screenings within 5 years for women aged 30–60. The results revealed significant statistical variations in at least one of these groups, highlighting disparities in healthcare delivery effectiveness across different levels of service transfer. These variations were significant, with *p*-values at 0.05, underscoring the profound impact of transfer intensity on health outcomes.

It also backs up what other research has found: big issues like trusting people’s beliefs and attitudes, dealing with information, and practical and logistical problems have a big impact on skilled providers’ ability to provide basic healthcare and deliver it to the people who need it. Despite these insights, the service providers’ report on these various aspects lacked clarity. Grut et al. [25] provided an example, demonstrating that health providers’ attitudes can act as significant barriers. After talking about outcomes, related issues, and how they affect the complicated and severe system of health-seeking processes, it seems that future efforts to boost service quality should focus on meeting the beliefs and needs of service recipients and figuring out how to make things work so that everyone can receive good healthcare.

We acknowledge several limitations in our study that should be considered when interpreting the findings. First, the study’s design is observational [12], which can introduce selection bias and limit our ability to establish causality between health service transfer and changes in health outcomes [23]. Second, while we have attempted to control for various confounders, residual confounding may still exist due to unmeasured or inadequately measured variables. Additionally, the specific dynamics and administrative structures of the health services involved in this study are unique to the settings we examined [4], which may limit the generalizability of our results to similar health system contexts. The reliance on self-reported data for some health indicators could also introduce response bias, potentially affecting the accuracy of these measurements. Finally, although informative, the statistical significance of some findings does not necessarily imply clinical significance, necessitating further studies to explore the practical implications of these outcomes in everyday healthcare settings [21].

## 5. Conclusions

### 5.1. Impact on Service Quality

The transfer of sub-district health-promoting hospitals to provincial administrative organizations has led to notable changes in service quality and outcomes. Our findings indicate that hospitals that underwent full transfers (100%) experienced marked declines in service quality across various performance indicators. These declines were significant and stood in contrast to the expected outcomes. Such transformations highlight the profound impact that administrative restructuring can have on healthcare delivery and underscore the need for careful evaluation of transfer policies and practices.

### 5.2. System Development and Nursing Practices

System development goals were generally met, but there were disparities in expectations and actual outcomes, especially in fully transferred groups. Nursing practices and role efficacy varied significantly, indicating both challenges and areas of effective performance post-transfer. The following key barriers to effective service delivery were identified: insufficient staffing, inadequate medical supplies, unlinked referral systems, and insufficient budgeting. These factors critically hindered the ability of hospitals to meet health service demands effectively.

**Implications for Practice**: Our findings highlight the significant changes in service quality and outcomes following the transfer of sub-district health-promoting hospitals to provincial administrative organizations. These changes underscore the critical need for these organizations to enhance strategic planning and resource allocation to support health services adequately. Establishing effective policy oversight and comprehensive service standards is essential to guiding operational processes and improving service delivery. Moreover, we should prioritize continuous professional development and training for nursing staff and other health professionals to bridge service provision gaps and elevate care quality. Increased community engagement and transparency about health service provisions can also help to align service delivery with local needs and expectations.

**Recommendations for Future Research**: The complex dynamics observed in this study suggest that future research should adopt longitudinal designs to track the long-term effects of hospital transfers on service quality and patient outcomes. Comparative studies examining different degrees of transfer—such as less than 50%, 50–99%, and 100% transfer—could shed light on more nuanced impacts on service quality and operational effectiveness. Additionally, qualitative research focusing on the experiences and perceptions of both service providers and recipients before and after hospital transfers would offer deeper insights into the challenges and opportunities presented by these policy changes.

In conclusion, the intent behind transferring health service management to provincial administrative organizations often aims to enhance efficiency and service delivery. However, the actual outcomes can vary significantly, influenced by multiple operational, cultural, and logistical factors. Addressing these challenges through informed policy decisions, strategic management, and continuous stakeholder engagement is essential for improving health outcomes in the community. This study serves as a foundation for both policymakers and administrators to reconsider and refine approaches to health service management transfer to ensure the benefits outweigh the potential disruptions.

## Figures and Tables

**Table 1:** Nurses’ perspectives on subdistrict health-promoting hospital services.

Health Service Missions	Group 1: Provincial Transfer	Group 2: More than Half Transferred	Group 3: Less than Half Transferred
Medical Treatment	Standardized Services	Standardized Services	- No Difference
Health Promotion	Meeting Standards	Meeting Standards	- No Difference
Disease Prevention and Control	Covering All Age Groups	Covering All Age Groups	- No Difference
Health Rehabilitation	Covering All Age Groups	Covering All Age Groups	- No Difference
Emergency Medicine	Increased Training	Increased Training	- No Difference
Palliative Care	Comprehensive	Comprehensive	- No Difference

**Table 2:** Comparison of outcome differences according to the indicators of primary agencies meeting targets, by transfer groups.

Indicators	Transfer Groups	Average Difference in Outcomes	SD	F	*p*-Value
1. Percentage of Thai population aged 35–74 years who have been screened for diabetes by measuring blood sugar levels.	Group 1	−14.61	21.28	6.52	0.002
	Group 2	−16.66	23.91		
	Group 3	−4.16	11.10		
2. Percentage of Thai population aged 35–74 years who have been screened for high blood pressure.	Group 1	−0.16	0.43	0.82	0.440
	Group 2	−0.23	0.47		
	Group 3	−0.22	0.42		
3. Percentage of pregnant women who had their first antenatal care visit within 12 weeks.	Group 1	−8.45	16.60	4.89	0.008 *
	Group 2	−4.11	19.34		
	Group 3	0.46	17.24		
4. Cumulative percentage of cervical cancer screening coverage in women aged 30–60 years within 5 years.	Group 1	−8.45	16.60	4.03	0.019 *
	Group 2	−4.11	19.34		
	Group 3	0.46	17.24		

* *p* < 0.05: Group 1: 100% transfer, Group 2: 50–99% transfer, Group 3: less than 50% transfer. Analyzed using ANOVA statistics.

## Data Availability

The data supporting the findings of this study are available from the corresponding author upon request.
